# Medication Adherence and Compliance: Recipe for Improving Patient Outcomes

**DOI:** 10.3390/pharmacy10050106

**Published:** 2022-08-28

**Authors:** Taiwo Opeyemi Aremu, Oluwatosin Esther Oluwole, Kehinde Oluwatosin Adeyinka, Jon C. Schommer

**Affiliations:** 1 Department of Pharmaceutical Care & Health Systems (PCHS), College of Pharmacy, University of Minnesota, 308 Harvard Street SE, Minneapolis, MN 55455, USA; 2 Division of Environmental Health Sciences, School of Public Health, University of Minnesota, 420 Delaware Street SE, Minneapolis, MN 55455, USA; 3 Division of Epidemiology & Community Health, School of Public Health, University of Minnesota, 1300 S. 2nd Street, Minneapolis, MN 55455, USA; 4 Department of Radiation Oncology, University College Hospital (UCH), Ibadan 200248, Nigeria

**Keywords:** medication adherence and compliance, medication nonadherence, patient outcomes, health outcomes, providers’ education, patients’ education, communication

## Abstract

The indices of patients’ health outcomes have historically included recurrence of symptoms, number of emergency visits, hospitalization and re-admission rates, morbidity, and mortality. As significant healthcare players, providers can influence these events, including the timeliness of diagnosis and disease management, the cost of treatment, access to health insurance, and medication adherence. Beyond healthcare availability and access, the ability of patients to adhere to providers’ treatment recommendations goes a long way to serve as a recipe for improving patient outcomes. Unfortunately, medication nonadherence has been prevalent, culminating in worsened health conditions, increased cost of care, and increased healthcare spending. This article provides some innovative ideas and good considerations for encouraging medication adherence. Improving providers’ and patients’ education and adopting active and passive communication, including consented reminders, could enhance compliance. Embracing partnerships between providers’ organizations and faith-based and community organizations could drive adherence. Adopting an income-based cap on out-of-pocket spending and adapting the physical properties, bioavailability, and dosage regimen of medications to accommodate diverse patient population preferences could encourage refills and compliance. Good medication adherence can culminate in improved patient outcomes.

## 1. Introduction

Beyond the glitz and glamor of advances in medical technology in the 21st century and the technicalities of drug development, well-informed prescribing, and medication dispensing, what happens to the patient is the ultimate ideal known as the patient’s outcome. Patients’ outcomes encompass the events surrounding illness, disease investigation, and medication use, including persistence, recurrence, and remission of symptoms, number of visits to the emergency room, hospitalization and readmission, timeliness, effectiveness, and safety of care, morbidity, and mortality. Some factors influencing patients’ health outcomes may include timeliness of diagnosis and disease management, cost of treatment, access to medical insurance, and medication adherence.

## 2. Medication Adherence and Nonadherence

Medication adherence, otherwise known as medication compliance, is a successor to treatment recommendations. Therefore, it can be defined as the “act or extent of conforming to a provider recommendation/prescription based on timing, dosage, and frequency of medication use” [1]. It can also be defined as “a ratio of the number of drug doses taken to the number of doses prescribed over a given time period” [2]. Medication compliance can be determined using the medication possession ratio (MPR), self-report adherence scale, pharmacy refill records and pill counts, micro-electric event monitoring, biological indices (blood or urine levels of drugs or its metabolites), and supervised dosing [3]. Medication compliance is a precursor of patients’ health outcomes. As such, nonadherence to medication can result in poor health outcomes, including worsening medical conditions, an increase in comorbidities, and death [4]. The resultant increase in health needs from medication nonadherence often culminates in an increase in the cost of care, which invariably increases overall healthcare spending [4]. Medication nonadherence is, therefore, an issue of global health importance. To proffer solutions, it is essential to understand some of the influencing factors, grouped into three: providers, patients, and medications.

### 2.1. Providers’ Factors

As one of the stakeholders of patient care, providers, including physicians, pharmacists, nurse practitioners, and physician assistants, play a role in determining whether patients comply with prescriptions or not. Due to the demand of daily routines, providers may get carried away focusing on disease dynamics and treatment options, neglecting to focus on patients’ acceptance of treatment modalities, especially when it involves using medications. This results in providers failing to adequately educate the patients about the formulation, timing, dosage, frequency, side effects, and costs of the prescribed medicine [5].

### 2.2. Patients’ Factors

Patients are the primary stakeholders of health care, hence the need to consider their needs while dealing with nonadherence to medications. While acknowledging that patients are custodians of their wellbeing, it is imperative to note that some deviations may be due to misinformation about their diagnosis and treatment options. Factors including illiteracy, polypharmacy (multiple medications), alcohol use, cultural issues, religious beliefs, and the paucity of knowledge about the effect of treatment options can adversely influence medication adherence [6,7,8]. Mental health issues beyond patients’ control, such as depression and cognitive impairment, can equally contribute to nonadherence [6,7]. The socioeconomic status of patients, a sequel to whether they are employed or unemployed, determines their access to health insurance and, consequently, their ability to afford their medications.

### 2.3. Medication/Treatment Factors

The medications’ characteristics, including the pharmaceutical formulation, dosage, size, frequency of use, and the dosage forms of the drug (for example, tablets, capsule, powder, suspension, emulsion, syrup, injection, aerosol, and foam), can influence adherence. Cost, timing, and side effects could also be potential barriers to adherence [7]. For example, the side effects of antiretroviral therapy (ART), including headaches, diarrhea, vomiting, and peripheral neuropathy, can discourage compliance to what is supposed to be a lifelong medication. Non-compliance to ARTs can result in increased viral load, reduced CD4 counts, and poor health outcomes.

## 3. Approaches to Improving Medication Adherence

### 3.1. Providers’ Education

The providers’ education can influence the patients’ ability to adhere to prescribed therapies/medications [9,10]. As catalysts for medication adherence, providers must be well-informed about the characteristics of the drug options available for the illness being managed. Routine hospital grand rounds and continuing medication education can actively focus on improving providers’ education. Health care teams can adopt a care protocol that includes discussing each drug option’s pros and cons. The cost of brand versus generic, side effects of the medications, and the number of times patients would use the medication daily should be considered as possible content of the providers’ study guide. An understanding of drug characteristics puts the providers in a position that identifies with the patients’ realities.

### 3.2. Communication

Beyond providers’ knowledge is a strong patient–provider relationship and providers’ ability to pass the message on to recipients (patients). A possession of communication and interpersonal skills permeated with empathy and acknowledgment of the patients’ challenge of using medications for acute and chronic diseases can foster compliance. By their proximity to the community, community pharmacists can facilitate a line of communication to patients with dignity, respect, and understanding as fulcrums [4,11]. Providers are instrumental in encouraging compliance by increasing awareness, serving as worthy role models, and sharing personal testimonies if any. They can foster compliance by adopting consented reminders via text messages, emails, automated calls, and weekly mailed letters. This measure can help mitigate the unintended effect of forgetfulness.

### 3.3. Patients’ Education

An informed patient population is a recipe for improving medication adherence. Knowledge about the implications of not adhering to the providers’ instructions on medication use can foster compliance. An understanding of the importance of attending the required clinic visits/routine follow-ups can equally enhance compliance. Delivery of these messages can be through any of the following:

#### 3.3.1. One-on-One Interaction with Healthcare Professionals

This could entail the historic word-of-mouth method of disseminating information. Messages are most likely to be accepted by patients if the messengers are healthcare professionals because they are often regarded as credible sources of health information [12]. To reinforce such information, handouts or pamphlets could be handed from providers to patients or caregivers in a one-on-one situation. This form of interaction that fosters patients’ education can occur during routine clinic visits and follow-ups that may occur either in person or remotely (telemedicine).

#### 3.3.2. Mass Communication Using Social and Digital Media

With the rising trends of misinformation and conspiracy theories about medication use on social media fueling medication nonadherence, health care professionals/providers bear the considerable responsibility of tactically refuting this misleading information. Patients should be aware that their providers and health departments/ministries (local, state, and federal) are credible sources of information. The respective providers’ local and national associations can be at the front line of educating the public through printed media (e.g., flyers, posters, billboards, and newspapers), social networking sites (e.g., Facebook, Twitter, and Instagram), text messages, mobile applications (e.g., WhatsApp and Telegram), blogs, websites, televisions, and radios [13]. Printed media materials can be accessible in publicly available stores selling or promoting health care products and services (e.g., pharmacies and drug stores). Free digital media materials can be easily accessible to individuals with access to computers, laptops, tablets, or cell phones.

#### 3.3.3. Community Organizations, Including Faith-Based Organizations

Studies have shown that families are usually willing to accept messages from community and faith-based/spiritual leaders [12,14]. These leaders are well-known and trusted community members. The providers’ national and local associations and allied bodies, including public health associations, can adopt an active partnership with community and faith-based organizations. This union can serve as a driver for improving the health knowledge base of members and followers.

### 3.4. Advocacy

The high cost of healthcare in the United States (US) and elsewhere can limit the ability of patients to afford and adhere to treatment options. Therefore, through their respective associations/bodies, providers can champion policy proposals that advocate a cap on out-of-pocket spending on all prescription drugs based on the income and socioeconomic status of the patient population served. Because the affordability of medications that puts less strain on patients’ income can improve refills and adherence, providers can educate elected officials about the importance of being at the forefront of improving health outcomes and how to enhance health outcomes. Providers can also encourage policymakers to enact well-informed health-related policies that focus on cost, availability, and access to prescription drugs.

### 3.5. Adaptation of Medications

While acknowledging the ease of medication access through medication synchronization programs, medication packaging, and delivery services in the US [15,16], a universal adaptation of these efforts could aid more robust patient outcomes due to the influence of global connectivity and population growth. Considering nonadherence owing to the physical properties of medicines, when possible, manufacturers can ensure that every drug has different formulations, such as a liquid or solid, to accommodate patients’ preferences. Manufacturers can adapt medications’ strength, bioavailability, and dosage regimens to accommodate a less frequent administration. For example, adopting slow-release capsules that patients can use once per week can encourage refills and compliance, especially in recipients of polypharmacy. Creating a capsule option of tablets and offering patients the opportunity to choose can enhance compliance. Because humans are uniquely different in desires, tastes, and wants, medicines can be created in different flavors, giving them an appealing appearance and boosting users’ morale. Globally, researchers can continue to work towards improving existing medications to include fewer side effects. Table 1 provides a snapshot of these approaches to enhancing medication adherence.

## 4. Conclusions

Patients’ adherence to medications is a recipe for improved health outcomes and overall wellbeing. With the continued existence of life, there will continue to be atypical conditions that negatively affect the body for which medications are designed as a corrective measure. While acknowledging factors contributing to nonadherence, providers, patients, and drug manufacturers should continue to explore innovative ways to encourage compliance. A well-rounded, informed, and defensive provider cohort with a well-informed patient population and diverse medication options can promote medication adherence, which invariably improves patients’ health outcomes.

## Figures and Tables

**Table 1:** Approaches to improving medication adherence.

Factors/Approaches		Components
Advocacy		Cap on out-of-pocket spending on prescription drugs. Educate elected officials on the health and economic implications of improving patients’ outcomes.
Communication		Adoption of consented reminders to use medications via text messages, emails, automated calls, and mailed letters. Empathy and respect. Good communication and interpersonal skills. Strong patient–provider relationship.
Medication adaptation		Adaptation of different formulations for each drug when possible. Adaptation of medications’ strength, bioavailability, and dosage regimens that accommodates a less frequent administration. Improve existing medications to include fewer side effects. Universal/global adaptation of medication synchronization programs, medication packaging, and delivery services.
Patients’ education	One-on-one interaction with healthcare professionals	Handouts or pamphlets. Word-of-mouth dissemination of information.
	Mass communication using social and digital media	Free digital media materials. Printed media, social networking sites, text messages, and mobile applications. Refute miscommunication and conspiracy theories.
	Community and faith-based organizations	Adoption of active collaboration between the providers’ associations/bodies and community (and faith-based) organizations.
Providers’ education		Adoption of care protocol that includes the pros and cons of each drug option. Provider study guide consisting of cost of brand vs. generic, dosage, mechanism of action, and side effects of medications.

## Abbreviations

MPR	Medication possession ratio
ART	Antiretroviral therapy
